# “First Catch Your Hare”: Some Difficulties with, and Contextual Factors in, Understanding (In)Appropriate Workplace Relationships

**DOI:** 10.3390/bs12050126

**Published:** 2022-04-27

**Authors:** Steve Duck

**Affiliations:** Department of Rhetoric, University of Iowa, Iowa City, IA 52242-1486, USA; steve-duck@uiowa.edu

**Keywords:** inappropriate relationships, triangulation, attribution, social power, workplace relationships, enemies, conflict, social relationships, personal relationships, relationship science, rhetoric

## Abstract

The article considers the contextual factors that lead to descriptions of workplace relationships as appropriate and inappropriate. It reviews viewpoint, context of activity, and the tension between social and personal relationships in environments based on task completion. If relationships are the sum of series of interactions, then interactions must be judged in context before compilation. The vantage point of viewers will complicate these assessments, as will the rhetorical purpose of the reporter.

## 1. Introduction

Managers usually forbid coworkers from conducting “intimate” relationships at work, and HR policies typically comprise constraints on personal relationships during worktimes and in workspaces [1]. Moral or other judgments such as this, about the instantiation of kinds of relationships through specific kinds of behavior necessarily import an assessment (by society or, more accurately, by its specific representatives, such as neighbors, friends, and workmates) about activity that goes further than mere classification of relationships or behaviors. It attaches to them as a perspective taken by an observer that classifies, but also interprets, both in any report that the observer gives. Judgments of (in)appropriateness, therefore, require special consideration not only as commentaries on behavior but also in relation to social norms and observers’ purposes [2]. We can legitimately ask, therefore, whether those investigating such “difficult” relationships have adequately defined their object before attempting to explain it and whether they have necessarily caught their hare before attempting to cook it.

Such questions raise a fundamental problem. While society approves of some kinds of intimacy as the highest pinnacle of commitment and even stipulates them as a duty (between spouses), the same acts between random strangers are evaluated differently [1]. What is regarded as loving at home is reported by outside observers as disgusting and problematic at work or in public. So, is it observation or the context that makes a difference (since it could not be the act itself but rather its symbolism or attributed significance, when it is observed in a particular place)? Why is it that researchers regard an act performed in one context (at home) as entirely suitable—and, indeed, dutiful and praiseworthy—when, in another context (at work), it would be despised and seen as utterly reprehensible (even if the spouses worked at the same place)?

One clear inference from these simple questions and observations is that no activity or behavior has an inherent value or morality [2], but that the assessment of behavioral qualities derives from the vantage point of an observer. A vantage point inherently makes a difference to moral judgments, since an act can be explained in terms of context rather than as an act in itself. In essence, an observer’s judgment about the value of an action is *imported to* the explanation of a behavior in context rather than being *derived from* it. 

Noting that personal workplace relationships (PWRs) have several different possible forms, Horan et al. [3] observe that employees have choices over the extent to which they establish personal closeness with other employees or whether they extend any lateral or hierarchical relationships in the workplace to a more recognizably special level of relationship. Mutual and consensual PWRs range in intimacy levels but are not inherently problematic unless interpreted that way by observers, whether these are outside or inside the subject relationship. For example, as Horan et al. [3] note, cross-sex relationships at work may be seen as romantic by outsiders, but the same behavior may be perceived as platonic by insiders, given that they both know that one of the partners is gay or lesbian. The relationship behavior itself does not prompt the judgment; rather, the judgment is justified by other assumptions imported into the explanation by a reporting observer (who may also be a relationship participant). What may be important here is the purpose and the personal knowledge of the observer in making a judgment or characterizing a relationship or the behaviors in it.

When applied to communication in relationships about difficult or controversial topics, this idea can be a foundation for approaches that focus on the different mindsets of the partners to approach a common problem that may be “located in” either one of them [4]. Research efforts to judge, assess, or classify couple communication without appreciating the larger social and economic contexts of those behaviors may be premature or counterproductive. 

## 2. Taking a View

From this point of view, it is not wise to characterize any specific behavior or act as having an inherent quality of negativity, difficulty, or inappropriateness, just as competitiveness at work may be judged as creditable (“go getter” and “can do attitude”) or as undesirable (“not a team player”). Rather, behaviors can be attributed to characteristics only relative to three factors: (1) the context of their occurrence; (2) the likely “location” of the origin of reports about behaviors; and (3) the purpose of the description, since the choice between points of explanation is inherently a motivated choice, one based on the intent of the observing reporter. 

### 2.1. Context as Interpretive Guide

Context may be everything in this definition, since a person may be in a legal or socially defined relationship with another person (e.g., spouses), but that relationship could also be described as intimate, disruptive, combative, competitive, loving, and unpredictable in the context of specific interactions. Although we do it all the time, it would be naive to describe any relationship with one persistent and exclusive label, as it would be to ignore the social moralizing that limits expressions of intimacy to specific contexts. 

However, any discussion of “relationships” assumes an easy answer to the question of what “a relationship” is. Researchers are not used to challenging questions like this and are normally content to assume that “somehow” a relationship is the sum of its interactions. This is not a safe assumption, for at least two reasons: 

(1) There is a clear distinction in the scholarly research on relationships between “social relationships” and “personal relationships” [5]; a **social relationship** is essentially a role relationship between two people who may be interchangeable without affecting the nature of the relationship, such as customer–cashier, or worker and production manager. In contrast, a **personal relationship** is immediately altered by the exchange of either party with someone else. It is obvious that, in “relationships at work”, there are two concurrent kinds of relationships that can be identified between two specific individuals in the workplace, one of them a role-based social relationship and the other a personal connection that is readily identified by the manner in which the social relationship is personally conducted. Horan et al. [3] represent an overlapping lens of two normally distinct conceptual circles and may be evaluated within the norms of either. The question is, “In a given instance, which set of norms might suggest (if not control) the assessment for an observer?” It is most common when the expectations (of a PR) influence the benefits (in an SR) that observers’ eyebrows are raised, as when a friend uses friendship, rather than business judgment, to decide in favor of a workmate. Then the decision is judged sleazy or corrupt (at least in the West; in the Middle East, contracts are expected to be awarded to relatives, and not necessarily to the highest bidder [6]).

(2) The second difficulty arises from the equally relevant question of how to derive a logical association between specific interactions and the judgment about the nature of a relationship (as an overall, and yet very much simplified, summative condensation of all these interactions). Even if a formal relationship exists in law or in social roles (as in, for example, “a marriage”), the emotive or experiential character of that relationship moment-to-moment is not derivable from the legal definition, or even its social definition. People in loving relationships go through periods of difficulty or dissension that may be part of the rich tapestry of that relationship in the long-term view. However, even researchers still tend to ascribe a long-term singular label that obliterates these variations in daily experience. In part, this summative oddity is unexplainable, and it is important that relational scientists have notably failed to define what a relationship may be [7]. It is more important here to take account of the fundamental importance of the purposes of a describer (including “relationship scientists”) in characterizing it in a preferred way. 

Similarly, people who are in a formal social-role-based relationship at work (e.g., as boss and employee or as two employees in the same place) can experience other kinds of daily interaction that may be formal, friendly, encouraging, or confrontational, but the overall and socially agreed label of “workplace relationship” would remain in place irrespective of the nature of specific interactions. Within that dull, summative definition from the vantage point of legal and social context, many excitingly differentiated emotional geographies will thus be erased. Actual interactions may better exemplify enemyship [8], or a distant social relationship may be delineated purely by its agentic functions, or a close personal friendship or even a romance. Once again, the nature of the formal definition of the social relationship between the two parties is not uniquely indicative of only one kind of personal interaction between them on a given occasion of observation or report. Nor is it truly an objective entity, since rhetoricians routinely observe that anything can be described in multiple (“copious”) ways, and a particular choice of description is shaped by those seeking to label it on a particular occasion for a specific audience or purpose.

Researchers need to take account of the fact that a range of types of interaction may occur within “the same” relationship, that a given interaction on a particular occasion may not represent the more usual style of interaction within that relationship, and that a characterization or description of it represents an interpretation by the observers (participants or outsiders). For example, in the workplace, an ambitious junior colleague might be seen as unduly pushy or as excitingly innovative if s/he makes frequent suggestions. These suggestions themselves may be favorably interpreted by friends but very negatively interpreted by rivals, not simply on the basis of their substance, but on the basis of each type of observer’s beliefs about the personal consequences of adopting them. The point, then, is that an assumption that any relationship has a simple, stable, and persistent “true label” is a mistaken one. It misdirects researchers to look for characteristics of “a relationship” rather than to explore the circumstances of a reporter commenting upon it in a particular way and the resources upon which he or she draws to do so.

The circumstances of reporters occur under a broader and vaguer umbrella where social norms and observer intent should be calculated into the analysis. In a broader review of such questions, Duck et al. [9] (p. 12) argued that “the essence of difficulty in relating is … better understood as strongly contextualized rather than as an inherent quality of persons or relationships”. Such contextualization may be found in the place of interaction, the norms, and the definitions that surround or characterize such a place, and the differences that observers recognize as constraints on activity and expression, whether in a formal or informal setting, in a ritual space (such as a church) or at a party. 

In this context, a PWR is both formally defined (as something occurring in a particular space) and formally or informally conducted at the personal level, but no single description of the relationship either defines or dictates the observer’s “copious” report. Such social relationships not only can be conducted on the personal level in either a friendly or negative way, while still populating the same social definition, but also can be described in a multitude of ways for different purposes on separate occasions (in a letter of reference, a formal report, a casual conversation, or a scientific article). The purpose of the description, however, interacts with the sort of description that is offered or accepted. Although scientists would very much like to see a relationship as a purely objective entity for their measurement, reality actually points in another direction. 

By seeing a workplace as a particular kind of space that has universal rules and norms that are not present in other less formal or task-oriented spaces, authors have assumed a uniformity of experience within it that may be illusory in key respects that affect their inquiries into “difficult relationships” there. Thus, while it is acceptable to be friendly in the workplace, too much friendly activity or chit-chat would be regarded as undermining the fundamental purpose of the workplace: namely, to get tasks completed. However, the exact exemplification of “friendly activity” or “chit-chat” may differ between observers, like many other observations. So, even if observers see a task-oriented requirement as a primary demand in the workplace, it does not follow that they would always agree on the nature of informality or that they would necessarily agree that a relaxed or friendly parallel relationship there would be inappropriate. Nor is agreement forced concerning the extent to which it might interfere with the performance of the predominantly expected activity, namely task advancement. Sias and Shin [10] have discussed this broadly, though Jung and Yoon [11] found that workers’ romances can *improve* commitment to the workplace and lead to *superior* performance of the job in the hospitality industry, as judged by inside observers.

So, what can make relationships at work “difficult”, or what can lead an observer to describe it as such? One obvious point is that, if we assume objectivity about relationships, then apparent tension between the social and personal definition of relationships might be identified as the same by all observers. This might lead one to ask if there are special tensions between the social and the personal aspects of relationships that are heightened or exaggerated by the “objective” location of the two individuals in the same workplace or instead by the position of the reporter. Alternatively, it might lead us to ask if the disjunction of norms in a situation places an observer in a bind as to which sort of description should be used to summarize or characterize the observation of what happens there.

### 2.2. “Location, Location, Location” 

Judgments and reports made about any behavior are influenced by an understanding of the circumstances in which it occurs, and which might offer partial or complete explanations for the action. For example, a person infringing upon another’s personal space may be excused if the behavior happens in a limited enclosure, such as an elevator, and is also seen as unintentional or “created” by the limited spatial arrangement. The same behavior may be classified as intrusive when unwanted by an insider, as in sexual harassment, but it, nevertheless, could be seen as exciting and intimate if it is desired by the same insider.

Foley [12] (p. 43) points out the problem of locating the source of “relational difficulties” and the extent to which it may be sited in a person, an interaction, social discourse, or a more complex systematic approach to the interaction of all of those elements. Explanations can also be seen in the context of larger human tendencies to explain observed behavior. The Fundamental Attribution Error [13] is too often overlooked in discussions of “difficult” behavior, but it testifies to the fact that observers/outsiders explain behavior in fundamentally different terms from those used by the insiders to explain their own behavior. Whereas persons themselves (inside observers) will point to the important situational features and stimuli in response to which their behavior was performed, outside observers tend to explain all the behavior of other people in terms of attributed personality characteristics or unifying themes that take little account of such situational influences. Whereas an inside observer might explain his or her “difficult” responses in terms of threat or provocation, an outside observer might be inclined to describe it in terms of characteristic irritability, difficulty, or pugnacity. Chory and Gillen Hoke [14] reported that workplace romances, especially hierarchically unequal ones, were seen more negatively by others than the participants themselves believed they would be and led to reduced trust and less honesty in communication in the workplace in general. 

### 2.3. Observers’ Reports and Their Framing

This latter finding adds to a growing sense that the mechanisms of reporting on observed relationships need fuller exploration. Reports of relationships are not so much logical alternatives as they are rhetorical choices that can be made by the parties involved. As Kenneth Burke [15] points out, there are five basic elements to any story: The Pentad of scene, act, agent, agency, and purpose. The decision to focus on any one of these specific elements will steer the interpretive possibilities that are chosen by observers. Thus, for example, in a case where students are found to be skipping classes, a focus on **agent** would tend to lead to the claim that the students are lazy; a focus on **scene** would tend to suggest that there are more attractive alternatives available to classes (such as a local beach); a focus on **agency** might point to the bus timetable on campus that limits the ability of certain students to get to the specific classes at a convenient time. 

The explanations for “difficult” behavior are likewise matters of observer choice rather than logical inevitability. It is the purpose of the speaker or observer that will urge one account rather than another upon the listeners. Assessments of others are not objective but are tinged with the intents of reporters rather than their status as insiders or outsiders, as the Fundamental Attribution Error oversimply has it. Hence, “difficult” relationships are partly defined as such by a describer and may not be inherently “difficult”. Thus, if we apply Burke to the generalities, we might need to review previous research in light of the ways in which insiders and outsiders make reference to agent or scene in explaining their “difficult” circumstances to others (as indicated by the Fundamental Attribution Error). More informatively, we might consider how different attribution of causes to agent, agency, or scene might be used not only as descriptors but as explanations of the observed events. Research might also explore the tendency of different types of observers to focus on certain aspects of the general circumstances and to place one element in a ratio relative to another (for example, a scene–act ratio explains the act in terms of the scene, as in “Desperate times call for desperate measures”).

When characterizing any behavior as inappropriate or difficult, therefore, one must first articulate the context in which that characterization is reported, and one must also attend to the purposes of the describer. Behaviors have no inherent qualities and can be labeled “inappropriate” only when all observers agree or when other explanations are ruled out or socially preferred (for example, if the breaching behavior was committed by a very young child who knew no better, or if a person said something but was known to be “lacking in social skill” rather than having malicious intent [16] or their secretiveness in times when self-disclosure is expected can be explained by shyness or anti-intraception [17]). 

If researchers have made a mistake in regarding relationships as composites of interactions [18], and if the description of a relationships is not absolute but is inflected by circumstances and by observers’ purposes, as well as by the occasion of the description, then research on problematic PWRs needs a recharge. 

The fact that two people work in the same environment repeatedly and have connected interactions, whether intimate or distant or transactional, may be enough for us to assume that they have some sort of relationship, even if, at this point, we are not able to give it a general label. Thus, the very fact that two people work in the same place makes them workmates, even though this “relationship” between them may not be the only one or even the most important. A son working for a mother in the family business is nonetheless in two different relationships, and tension may arise in some circumstances more than others. “Relationships at work” may therefore be more than just the relationships of workmates in the same space, and difficulties may arise from role strain rather than from inherent causes within the workmate relationship [19]. 

Given that background, it is safe to assume that relationships at work are considered as essentially a continuation of other kinds of relationships but within a special and circumscribed context (for example, what counts as adultery depends on whether a society allows polygamy). Context may influence an observer’s characterization or agreement about a behavior or a relationship, but a final important element is ultimately the describer, analyst, or framer, whether an insider of the relationship or an outside observer. 

## 3. Triangulation 

Duck et al. [9] (p. 226) indicated that a common feature of all examples of difficulty in relationship is triangulation: this involves the observation by one party of a kind of interaction between two other parties (also, in other ways, observers) and the way in which this triangle influences descriptions of any behavior. Whatever the two relaters themselves may say about an interaction or a relationship, the fact that it may be framed by an outside observer as “problematic” essentially makes it so, at least for some purposes. It is then sufficient for a framer to identify one of the relators as a source of a problem, even though either of the relators may dispute the fact. Carson [20] has recently elaborated on this idea by differentiating external relational attributions and external non-relational attributions, the former being attributions that locate the cause of behaviors within the relationship between two people and the latter being attributions that locate the causes elsewhere. However, as the notes on Burke above suggest, there are other possibilities for a reporter to use to position a report about “a relationship” and to attribute its features not to agents but to scenes or to other elements of the Pentad.

If observers focus on scene, then a reference to exterior social norms may be a key point of their description. If they focus on agents, then the norms will be given less prominence. However, observers, whether insiders or outsiders, are neither random nor error-prone deviants from reality; the association of behavior with a label is not simply semantic but partly cultural. It assumes deference to norms or cultural practices in particular spaces, even if those norms are not always a guide to actual practices. Thus, sexual harassment is often defined in terms of the superior power of the harasser over the harassed, and bullying is defined in terms of misuse of formal power in an informal context [21]. However, the attributions each depend on a reporting observer acknowledging the use of power and attributing its (mis)use to agents according to norms, rather than to scenes or to stress the role of the agent’s character (as more powerful, in ratio to the scene) in the production of the act.

Social Power at work, however, is not a simple concept [22], but power is often seen as illegitimate when not aligned with observed competence in agents. Leadership may be shown as management of relationships of unequal power to create a group that can effectively address a wide variety of problems, especially in the workplace. For these reasons, the operation of power (agency) in a workplace (scene) is partly a social observation rather than an objective reality, as is the attribution that it might engender conflicts by virtue of the nature of the task [23]. For example, the conduct of a personal relationship between two workers may be a result of perceived competition and aggressiveness that are evoked by the requirements and the norms of the workplace, rather than by individual preference and desire. Likewise, industrial sabotage and bloody-minded resistance to authority (agency) must be seen in contrast to social norms (scene) that presuppose politeness, cooperation, and willing collaboration with the demands of the workplace and its authority patterns. Thus, it is not seen as prejudicial to have positive relationships with some co-workers and not others if this does not interfere with the ability to get the job done. It is the social reference point and understanding of the context by an observer (who may also be a participant) that gives the description of the relationship or behavior its flavor.

Should we therefore regard workplace context and its norms as the essential influences on observers for deciding what is inappropriate and appropriate there, or do we need to do more research to discover what potential influences actually inflect reports? The very acceptance begs the question: in order to accept something as inappropriate in that circumstance, we have to presuppose what appropriateness to the situation amounts to in the eyes of a reporter, whether an insider or an outsider. Labels about behavior and intention depend not simply on behavior but usually by reference to some social rule or norm against which a perceiver assesses the behavior and their relationship.

A triangulator-as-framer necessarily has difficulty understanding the relationship between the two relators, but pronouncements about the relationship are difficult without full understanding of the interior of those interactions; however, such an individual may be in a better position to judge a relationship as abusive (for example) than either partner is. A triangulator can be an interference (for example, an in-law [24] or a non-residential parent [25]), a linchpin (a shy person’s social lubricator [26]), or a comparison point (a present person for a long-distance partner [27]), because difficulty is a process not an event. Perceived difficulty has to be managed by somebody, whether by changing their perceptions of the triangulator or modifying the behavior of the two relators. Without the perspective of the triangulating framer, relationship difficulty at work or otherwise is merely behavior. Either party can make it problematic by referring to a triangulation point, which may be the direct observations of others or the indirect and implied involvement of some framer, such as common social experience or norms.

We must abandon the notion that it is possible to describe “a relationship” objectively; since a relationship is a social construct, not an objective reality, descriptions and identifications of relationships and their characteristics must necessarily be tied up with the social context in which they are described by an observer with a particular purpose. Although researchers have assumed that it is possible to describe the relationship objectively and to indicate deviations from “reality” represented by one or other partner’s descriptions, this has long been condemned as naive [28]. The fact that two partners in the same relationship may describe it differently is not evidence of error but of the fact that the social construct is never identified merely by the agreement of its two constituents, and far less is it identifiable by the firm pronouncements of an outsider in an elusive search for agreement between the outsider and the insiders. Thus, the phenomenon that we identify as “difficult PWRs” must be measured in a different and more complicated way than by simply accepting the mere declarations of an observer, especially if that observer claims scientific (objective) credentials.

Given this, and despite its frequency of casual experience in everyday life, there is significance to the available range of “copious” descriptive opportunities for an entity that researchers would like to see as a single and universally defined “object”. The range and inconstancy have been overlooked theoretically, except by rhetoricians. Empirical research on theoretical observations about the importance of recognizing that even individuals in a relationship may differ from one another in this characterization has repeatedly failed to influence relationship researchers in their intent to regard “relationships” as objective and scientifically measurable entities. This scientific leaning toward objectivism simply ignores the data. Different observers characterize “the same” relationship differently, and this has been tacitly recognized for 40 years [28], but not openly or theoretically acknowledged in any real way [8]. The desire to regard relationships as scientifically objective has not yet recognized the importance of an observer’s viewpoint when characterizing, for particular social purposes, the “object” that is being described. Once this point has been accepted, then fruitful discussion of PWR difficulties can be equally subject to proper, perhaps even scientific, scrutiny, but first we must follow Hannah Glasse’s famous cookery advice in her recipe for Jugged Hare: “First catch your hare”.
